# The burden of disease on HIV-infected orphaned and non-orphaned children accessing primary health facilities in a rural district with poor resources in South Africa: a cross-sectional survey of primary caregivers of HIV-infected children aged 5–18 years

**DOI:** 10.1186/s40249-015-0049-x

**Published:** 2015-05-04

**Authors:** Mathildah M Mokgatle, Sphiwe Madiba

**Affiliations:** Department of Biostatistics, School of Public Health, Sefako Makgatho Health Sciences University, P O Box 215, Medunsa, South Africa; Department of Environmental and Occupational Health, School of Public Health, Sefako Makgatho Health Sciences University, Medunsa, South Africa

**Keywords:** Primary level of care, Hospitalization, Provider-initiated testing and counseling, Perinatally infected older children, Burden of disease, Orphanhood, South Africa

## Abstract

**Background:**

Provider-initiated HIV testing and counseling (PITC) is offered as part of the normal standard of care to increase access to treatment for HIV-infected children. In practice, HIV diagnosis occurs in late childhood following recurrent and chronic infections. We investigated primary caregivers’ reported reasons for seeking HIV testing for children aged 5–18 years, determined the orphan status of the children, and compared the clinical profile and disease burden of orphans and non-orphans.

**Methods:**

This was a cross-sectional survey of primary caregivers of HIV-infected children accessing antiretroviral treatment (ART) from two community hospitals and 34 primary healthcare facilities in a rural district in Mpumalanga province, South Africa.

**Results:**

The sample consisted of 406 primary caregivers: 319 (78.6%) brought the child to the health facility for HIV testing because of chronic and recurrent infections. Almost half (n = 183, 45.1%) of the children were maternal orphans, 128 (31.5%) were paternal orphans, and 73 (39.9%) were double orphans. A univariate analysis showed that maternal orphans were significantly more likely to be older (OR = 2.57, *p* = 0.000, CI: 1.71–3.84), diagnosed late (OR = 2.48, *p* = 0.009, CI: 1.26–4.88), and to start ART later (OR = 2.5, *p* = 0.007, CI: 1.28–4.89) than non-orphans. There was a high burden of infection among the children prior to HIV diagnosis; 274 (69.4%) presented with multiple infections. Multiple logistic regression showed that ART start age (aOR = 1.19, *p* = 0.000, CI: 1.10–1.29) and time on ART (aOR = 2.30, *p* = 0.000, CI: 1.45–3.64) were significantly associated with orphanhood status. Half (n = 203, (50.2%) of the children were admitted to hospital prior to start of ART, and hospitalization was associated with multiple infections (OR = 1.27, *p* = 0.004, CI: 1.07–1.51).

**Conclusions:**

The study found late presentation with undiagnosed perinatal HIV infection and high prevalence of orphanhood among the children. The health of maternal orphans was more compromised than non-orphans. Routine PICT should be strengthened to increase community awareness about undiagnosed HIV among older children and to encourage primary caregivers to accept HIV testing for children.

**Electronic supplementary material:**

The online version of this article (doi:10.1186/s40249-015-0049-x) contains supplementary material, which is available to authorized users.

## Multilingual abstracts

Please see Additional file 1 for translations of the abstract into the six official working languages of the United Nations.

## Background

Provider-initiated testing and counseling (PITC) has been advocated as a strategy to increase access to treatment and care interventions for HIV-infected children [1], but there is evidence that millions of children living with HIV remain undiagnosed or present late in the course of their disease to healthcare facilities [2,3]. Delayed diagnosis of HIV in perinatally infected children puts them at high risk of severe morbidity and mortality [4]. In South Africa, less than half of children in need of antiretroviral treatment (ART) have access to it [5]. Ramirez-Avila and colleagues argue that the World Health Organization’s (WHO’s) recommendations for PITC for children do not offer implementation guidelines [6]. On the other hand, efforts to identify undiagnosed HIV-infected children, outside of the prevention of mother-to-child transmission (PMTCT) program, are inadequate and also lack implementation strategies [2]. Implementation of PICT requires clear guidelines, including disclosure to children, age of consent for HIV tests, and care for older children and adolescents [7]. Nevertheless, offering PICT to sick children who present in health facilities is feasible and is an effective approach to identify undiagnosed HIV among older children [7,8].

While guidelines for the implementation of PICT are necessary for its success, parents and caregivers are crucial in the adoption of PICT for HIV testing of exposed or undiagnosed children whose mothers did not receive PMTCT interventions. However, literature shows that parents and caregivers are reluctant to have their children tested for HIV, and their reluctance poses a barrier for children to access HIV treatment [2,4]. HIV-related stigma has been shown to be more important in the decision-making process of parents and caregivers to test their children than the benefits of an HIV test. This is common among parents who are receiving ART themselves even when they suspect that their children are HIV-infected [4,6,9-11]. Strategies to increase children’s access to HIV testing must include interventions to address prevalent community attitudes and create awareness of the risk of HIV infection in older children [4]. Limited availability of testing services for children, difficulties in identifying HIV-infected infants, and poor referral networks have also been identified as barriers for HIV testing for children [3,7,12]. What compounds the problem is that HIV testing of older children in primary health facilities has been introduced only recently because the survival of perinatally infected children in the absence of treatment was thought to be uncommon [13].

As a result, for many children and adolescents, HIV diagnosis occurs in late childhood following many years of ill health [2-4]. Although the WHO recommends that in generalized HIV epidemics, like in South Africa, PICT should be offered as part of the normal standard of care [1], in practice, HIV testing relies on children having HIV-related symptoms before treatment initiation [3,4]. Consequently, there is a lack of empirical data on when and why older children access primary health facilities for HIV testing and counseling. There is also a paucity of data on the burden of opportunistic infections in HIV-infected children in poor-resource settings [14]. Most HIV prevalence surveys either exclude older children [4], or report on children less than five years of age [15,16]. This is despite evidence that up to a third of HIV-infected children are slow progressors with a median survival of 10 years [17], and make up about 3% of all undiagnosed perinatally infected 10-year-olds in South Africa [18]. These data justify extensive case finding strategies to identify these children for early initiation of treatment, care, and support.

In view of the reported barriers to HIV testing of older children in primary health facilities, it is likely that the true burden of HIV-related infections in undiagnosed children in poor-resource settings is unknown [14]. On the other hand, the impact of HIV infection on the clinical wellbeing of orphaned children is under investigated. There is also limited data on the number of orphaned children accessing HIV testing, care, and support in primary health facilities in rural settings. The focus of most studies conducted among orphans is mainly on the social wellbeing, educational attainment, psychosocial support, and caregiver experiences of caring for orphaned children [19-22]. We investigated parents/primary caregivers’ reported reasons for seeking HIV testing and counseling for children aged 5–18 years and accessing HIV care in primary health facilities in a rural district in Mpumalanga province, South Africa. We also determined orphan status and compared the clinical profile and disease burden of the children. Previous studies assessing HIV testing and counseling have been hospital-based, however we conducted the study in primary healthcare facilities in a rural district. In South Africa, ART for adults and children is currently accessible at the primary healthcare level through the Nurse-initiation and maintenance of antiretroviral therapy (NIMART) [23]. This initiation has increased access to HIV care and support for many communities. The study findings will provide insight into community awareness on late presentation of HIV in children and also inform the methodology for HIV testing and counseling for children.

## Methods

This was a cross-sectional study of primary caregivers accompanying children aged 5–18 years for ART treatment, care, and follow-up in primary healthcare facilities in the Nkangala district, Mpumalanga province, South Africa. A primary caregiver was defined as an adult who lives in the same house with the child, is responsible for the day-to-day care for the child, and has knowledge about the child’s HIV diagnosis, ART treatment, and care.

The study was conducted in a predominantly rural district between June and September 2013. Data were collected in two community hospitals and 34 primary healthcare facilities, of which seven where primary health centers (PHC) and 27 were eight-hour primary health clinics. The health facilities were located in three sub-districts: one urban and two rural. The majority of the health facilities, including the two district hospitals, were located in the rural sub-districts. These sub-districts are characterized by unemployment and poverty, but with adequate access to primary healthcare services, as the majority of people walk to the local primary health clinic or community health center. The catchment area of the urban sub-district included urban townships, informal settlements, and mining communities. In South Africa, patients are able to access counseling, testing, and treatment from primary healthcare facilities through PICT. The goal of the PICT is to increase the number of sites providing ART at the primary healthcare level through the NIMART initiative. The HIV services provided through NIMART include adult and pediatric counseling, testing, and initiation of ART. Nurses are trained to use the WHO clinical staging to assess children for eligibility for ART in the absence of CD4 count results [24]. The WHO clinical staging is used to determine eligibility for ART in poor resourced settings in which CD4 testing is limited. Clinical stages are categorized as stage 1 or asymptomatic, stage 2 or symptomatic, stage 3 or severe symptomatic, and stage 4 or advanced symptomatic. These guidelines recommend that ART should be initiated in all children infected with HIV with severe or advanced symptomatic disease regardless of age and CD4 cell count. The purpose of the revised guidelines was to align clinical and immunological criteria for ART eligibility for children older than five years with those for adults [25]. Nurses working in PHC facilities were trained to initiate ART before those working in eight-hour clinics, and this was evident during data collection where a large number of primary caregivers were recruited from PHC facilities.

Data were collected using interviewer-administered questionnaires. Seven trained field workers conducted the semi-structured interviews with the primary caregivers of HIV-infected children. The primary caregivers were eligible for the study if they were above 18 years of age and were biological parents or primary caregivers of children aged between 5 and 18 years who were receiving ART in the health facilities at the time of the survey. The face-to-face interviews were conducted during the clinic visits when caregivers accompanied their children for treatment. The clinic staff played a crucial role in the recruitment of caregivers who met the inclusion criteria by contacting and reminding them of their clinic appointments. In cases where older children collected their own medication, the community workers attached to the clinic contacted the caregivers and made appointments for the interview to be conducted either at the clinic or at home. As already stated, the majority of people walk to the local healthcare facility and it was feasible to conduct interviews in the participants’ homes. The questionnaires were prepared in IsiZulu, Sepedi, and English, and the primary caregivers were interviewed in the language of their choice. The children’s clinical records were reviewed to collect the WHO clinical staging data prior to diagnosis; initial and current CD4 count results expressed as absolute values; and children’s demographic information including age at HIV diagnosis, age at initiation of ART, and HIV-related infections that the child presented with at ART initiation. Information collected through the interviews included the demographics of the primary caregivers, the orphan status of the children, HIV symptoms, presenting infections, and the number of hospital admissions prior to HIV diagnosis and initiation of ART. One of the authors (MM) oversaw the training of field workers and implementation of the fieldwork, as well as ensuring data quality.

Ethical clearance was obtained from the Research and Ethics committee of the University of Limpopo, Medunsa Campus (MREC/H/168/2012: IR). The research committee of the Mpumalanga Provincial Department of Health and the district health office also gave permission to conduct the study. Informed consent was obtained from all the primary caregivers; they were informed that participation was voluntary and assured of the confidentiality of their responses.

Statistical analyses were performed using STATA version 13.0 (StataCorp, Texas, USA). The chi-square test was used to assess association between categorical variables and independent variables. Continuous variables were compared using the Student’s *t*-test, and descriptive statistics such as mean, standard deviation, and range were used for the continuous variables. Univariate and multivariate analyses using stepwise regression analysis were used to investigate the socio-demographic characteristics of the children associated with gender, maternal orphan status, and multiple infections. All the variables that showed significant association (*p* ≤ 0.005) at the univariate analysis were included in the stepwise logistic regression model. The maternal orphan status was used for analysis because the data on paternal orphan status was in some cases reported as unknown. Results are expressed as odds ratios (ORs) with 95% confidence intervals (CIs) and *p*-values. For all analyses, a *p*-value of less than or equal to 0.05 was considered significant.

## Results

### Demographic characteristics of primary caregivers

Four hundred and six primary caregivers of HIV-infected children aged 5–18 years were interviewed. The majority (n = 372, 91.6%) were females, the mean age was 44.2 years (range 17–89 years), 98 (24.1%) had a primary education, 163 (40.2%) had a secondary education, and 69 (17%) completed 12th grade. Almost three quarters (n = 280, 69%) were unemployed and 53 (13.1%) were elderly pensioners. The source of income for 282 (70.7%) of the primary caregivers was the child support grant and for 78 (19.2%) it was the old age pension grant. The HIV status of almost half (n = 194, 47.8%) of the primary caregivers was positive, 174 (42.9%) were negative, while the status of 38 (9.4%) was unknown (see Table 1).

**Table 1:** **Demographic characteristics of caregivers of HIV-infected children accessing primary health facilities according to child orphan status**

**Variable**	**Non-orphans (n = 223)**	**Orphans (n = 183)**	**All**	**OR/Con. interval**	***p-value***
***Caregiver***					***0.000***
Mother	158 (72.0)	0	158 (38.9)	1	
Father	4 (1.8)	7 (3.8)	11 (2.7)	0.003 (0.00–0.03)	
Grandparents	30 (13.5)	92 (50.3)	122 (30.0)	0.007 (0.00–0.01)	
Foster parent	4 (1.8)	8 (4.4)	12 (3.0)	0.003 (0.00–0.03)	
Other relatives	28 (12.6)	75 (41.0)	103 (25.4)	0.002 (0.00–0.01)	
***Gender***					***0.001***
Female	213 (95.5)	159 (86.9)	372 (91.6)	1	
Male	10 (4.5)	24 (13.1)	34 (8.4)	3.21 (1.49–6.91)	
***Age category (n = 404)***					***0.000***
≤40 years	142 (64.3)	56 (30.6)	198 (49.0)	1	
>40 years	79 (35.6)	127 (69.4)	206 (51.0)	4.07 (2.68–6.19)	
Mean age 44.2 (range 17–89 years)					
***Marital status***					***0.035***
Never married	125 (56.1)	78 (42.6)	203 (50.0)	1	
Married	66 (29.6)	71 (38.8)	137 (33.7)	1.72 (1.11–2.67)	
Divorced	9 (4.0)	10 (5.5)	19 (4.7)	1.78 (0.69–4.57)	
Widowed	14 (6.3)	20 (10.9)	34 (8.4)	2.28 (1.09–4.79)	
Living with partner	9 (4.0)	4 (2.2)	13 (3.2)	0.71 (0.21–2.39)	
***Employment status***					***0.000***
Employed	1 (0.5)	0.0	1 (0.2)	1	
Part-time employment	34 (15.3)	24 (13.1)	58 (14.3)	1.74 (5.33–5.65)	
Self-employed	8 (3.6)	6 (3.3)	14 (3.4)	1.63	
Unemployed	169 (75.8)	111 (60.7)	280(69.0)	1.87 (6.30–5.52)	
Pensioner	11 (4.9)	42 (23.0)	53 (13.1)	3.21 (9.20–1.12)	
***Educational status***					
None	23 (10.3)	37 (20.2)	60 (14.8)	1	***0.000***
Primary	35 (15.7)	63 (34.4)	98 (24.1)	1.11 (0.57–2.17)	
Secondary	107 (48.0)	56 (30.6)	163 (40.2)	0.32 (0.17–0.60)	
Completed 12th grade	47 (21.1)	22 (12.0)	69 (17.0)	0.29 (0.14–0.60)	
Tertiary	11 (4.9)	5 (2.7)	16 (3.9)	0.28 (0.08–0.91)	
***Income (n = 399)***					***0.000***
Child support grant	171 (78.1)	111 (61.8)	282 (70.9)	1	
Old age pension grant	23 (10.5)	55 (30.6)	78 (19.2)	3.68 (2.14–6.33)	
Other sources	25 (11.4)	14 (7.9)	39 (9.8)	0.86 (0.42–1.73)	
***Previously tested for HIV***					***0.000***
No	11 (4.9)	27 (14.8)	38 (9.4)	1	
Yes	212 (95.1)	156 (85.3)	368 (90.6)	3.33 (1.60–6.92)	
***Results of HIV testing***					***0.000***
Negative	46 (20.6)	128 (70.0)	174 (42.9)		
Positive	166 (74.4)	28 (15.3)	194 (47.8)	16.4 (9.77–27.8)	
Unknown	11 (4.9)	27 (14.8)	38 (9.3)	1.13 (0.52–2.46)	

### Orphan status of the children

Based on the primary caregivers’ report, almost half (n = 183, 45.1%) of the children under their care were maternal orphans, slightly over a third (n = 123, 31.5%) were paternal orphans, and 73 (39.9%) were double orphans. A significant number (n = 158, 38.9%) of the children were brought to the health facilities by their mothers; 122 (30.1%) by their grandparents; 103 (25.3%) by other relatives including aunts, uncles, and older siblings; 12 (3%) by foster parents; and 11 (2.7%) by their biological fathers. A significant proportion (n = 156, 86.2%) of the primary caregivers reported that they knew the cause of death of the biological mother of the child under their care. Of the 156 who knew the cause of death, 141 (91%) reported that it was AIDS-related. With regards to the cause of death of the biological father, 93 (73.2%) knew the cause of death and 78 (83.9%) attributed it to AIDS-related illnesses. The majority of maternal orphans have been orphaned an average of four years (see Table 2).

**Table 2:** **Orphan status of HIV-infected children accessing primary healthcare facilities**

**Variables**	**Number**	**Percent**
***Orphan status***		
Maternal	183	45.1
Paternal	128	31.5
Double (n = 183) *	73	39.9
***Father sees child***		
No	255	63.0
Yes	150	37.0
***Know maternal cause of death***		
Yes	156	86.2
No	25	13.8
***Cause of maternal death***		
AIDS-related	141	91.0
Other causes	14	9.0
***Know paternal cause of death***		
Yes	93	73.2
No	34	26.8
***Cause of paternal death***		
AIDS-related	78	83.9
Other causes	15	16.1

*The total percentage on orphan status does not add up to 100% because double orphans are calculated from the maternal and paternal orphans.

Of 183 children who were maternal orphans, half (n = 92, 50.3%) were cared for by their grandparents, 75 (41%) by other relatives, and 7 (3.8%) by their fathers. The average time maternal orphaned children had been under the care of primary caregivers was 6.9 years. For children whose fathers were alive, 150 (37%) saw their fathers regularly. This proportion of fathers includes those who were married (33.7%), which indicates that only 4% of maternal orphaned children see their fathers regularly (see Tables 1 and 2).

Logistic regression analysis showed that the primary caregivers of maternal orphans were likely to be less educated (OR = 0.60, *p* = 0.000, CI: 0.48–0.73) and almost four times more likely to depend on the pension grant (OR = 3.68, *p* = 0.000, CI: 2.14–6.33) compared to primary caregivers of non-orphans. The data further showed that caregivers of maternal orphans were four times more likely to be older (OR = 4.07, *p* = 0.000, CI; 2.68–6.19) than those of non-orphans. The results of the multivariate analysis showed no significant association between paternal orphans and the primary caregiver’s educational level, source of income, or age.

### Demographic and clinical characteristics of children

The characteristics of the children at the time they were diagnosed with HIV are presented in Table 3. Of the 406 children surveyed, over half (n = 210, 51.7%) were boys, 208 (51.3%) were aged 10–17 years, and 198 (48.8%) were aged 5–9 years; the mean age was 9.6 years. There were no significant differences between the mean age of boys and girls (M = 9.8, SD 3.2 years versus M = 9.5, SD 2.8 years). But when we compared the mean age of maternal orphans and non-orphaned children, there were significant differences; orphaned children were significantly older (M = 10.5, SD 3.0 years) than non-orphaned children (M = 9.0, SD 2.9 years).

**Table 3:** **Demographic and clinical characteristics of HIV-infected children accessing primary health facility, by orphan status**

**Variable**	**Non-orphans (n = 223)**	**Orphans (n = 183)**	**All**	***p-value***
***Age group***				***0.000***
5–9 years	132 (59.2)	66 (36.1)	198 (48.8)	
10–17 years	91 (40.8)	117 (63.1)	208 (51.2)	
Mean age 9.6 years (range 5–17 years)				
***Gender***				***0.386***
Girl	112 (50.2)	84 (45.9)	196 (48.3)	
Boy	111 (49.8)	99 (54.1)	210 (51.7)	
***Attending school***				***0.687***
No	9 (4.0)	6 (3.3)	15 (3.7)	
Yes	214 (96.0)	177 (96.7)	391 (96.3)	
***Age at HIV diagnosis***				***0.027***
0–5 years	125 (57.3)	85 (48.6)	210 (53.4)	
6–10 years	77 (35.3)	63 (36.0)	140 (35.6)	
11–16 years	16 (7.3)	27 (15.4)	43 (10.9)	
Mean age 4.8 years				
Missing values = 13				
***Age at ART initiation***				***0.023***
0–5 years	111 (51.4)	73 (41.7)	184 (47.1)	
6–10 years	88 (40.7)	74 (42.3)	162 (41.4)	
11–16 years	17 (7.9)	28 (16.0)	45 (11.5)	
Mean age 5.6 years				
Missing values = 15				
***Period receiving ART***				***0.146***
0–5 years	166 (76.2)	122 (68.9)	288 (72.9)	
6–10 years	48 (22.0)	47 (26.6)	95 (24.1)	
11–16 years	4 (1.8)	8 (4.5)	12 (3.0)	
Mean time 4.0 years				
Missing values = 11				

The mean age of the children at HIV diagnosis was 4.8 years (range 0–16 years). Slightly over half (n = 210, 53.4%) were diagnosed between 0–5 years, 140 (35.6%) between 6–10 years, and 43 (10.4%) were older than 10 years. This indicates that almost half (n = 183, 46.6%) were older than five years when they were diagnosed. There were no significant differences between the mean diagnosis age of boys and girls (M = 5.1, SD 3.8 years versus M = 4.5, SD 3.4 years). When we compared maternal orphaned and non-orphaned children, there was a significant difference between the mean diagnosis age; orphaned children were diagnosed late (M = 5.5, SD 3.8 years) compared to non-orphaned children (M = 4.3, SD 3.4 years).

The mean age at the time of ART initiation was 5.6 years (range 0–16 years). Less than half (n = 184, 47.1%) were initiated on ART between 0–5 years, 162 (41.4%) between 6–10 years, and 45 (11.5%) above 10 years. Of the 184 children initiated on ART between 0–5 years, only 16 (4.1%) were below one year. Over half (n = 207, 52.6%) were older than five years when they were initiated on ART. There were no significant differences between the mean age at ART initiation of boys and girls (M = 5.3, SD 3.3 years versus M = 5.8, SD 3.5 years). We found that maternal orphaned children were also older at the time they initiated ART (M = 6.3, SD 3.5 years) than non-orphans (M = 5.0, SD 3.2 years). Three quarters (n = 288, 72.9%) had been receiving ART for 1–5 years, 95 (24.1%) for 6–10 years, 12 (3%) for 11–13 years, and only 26 (6.6%) were on ART for less than one year. The mean time on ART was 4.0 years (range 0–13 years).

Logistic regressions analysis showed that maternal orphaned children were significantly more likely to be older (OR = 2.57, *p* = 0.000, CI: 1.71–3.84), to be tested and diagnosed late (OR = 2.48, *p* = 0.009, CI: 1.26–4.88), and to start ART later (OR = 2.5, *p* = 0.007, CI: 1.28–4.89) than non-orphans (see Table 4). At multiple logistic regression, using stepwise regression, ART start age (aOR = 1.19, *p* = 0.000, CI: 1.10–1.29), and time on ART (aOR = 2.30, *p* = 0.000, CI: 1.45–3.64) remained significantly associated with orphanhood, while multiple infections were not associated with any of the sociodemographic variables of the child.

**Table 4:** **Logistic regression analysis of orphan status, child demographics, and clinical characteristics**

**Orphanhood**	**Odds ratio**	**Std. err.**	**z**	**P > z**	**[95% Conf. interval]**	
Child age	2.57	.528	4.59	0.000	1.71	3.84
Hospitalization	0.82	.164	−0.99	0.322	0.55	1.21
Diagnosis age	2.48	.857	2.63	0.009	1.26	4.88
WHO clinical staging	1.14	.304	0.52	0.602	0.68	1.93
ART start age	2.50	.857	2.68	0.007	1.28	4.89
Time on ART	2.72	1.697	1.60	0.109	0.80	9.24
Multiple infection	0.53	.144	−2.30	0.021	0.31	0.91

### Reasons for seeking HIV counseling and testing for children

Primary caregivers gave reasons why the child was brought to the health facility for HIV testing and counseling. The majority (n = 319, 78.8%) reported that the child suffered from recurrent and chronic illnesses, 39 (9.6%) of the children were tested because one or both parents were ill, and 23 (5.7%) were tested after the death of the mother (see Table 5).

**Table 5:** **Reasons for testing, number of infections, WHO clinical staging, and hospitalization of children at diagnosis**

**Variable**	**Non-orphans (n = 223)**	**Orphans (n = 183)**	**All**	***p*** **-value**
***Reasons for HIV testing***				***0.000***
Child was ill	188 (84.7)	131 (71.6)	319 (78.8)	
Illness of parents	20 (9.0)	19 (10.4)	39 (9.6)	
Death of mother	0 (0.0)	23 (12.6)	23 (5.7)	
Other	14 (6.3)	10 (5.5)	24 (5.9)	
Missing value = 1				
***Number of presenting infections***				***0.115***
Single infection	59 (49.9)	60 (50.4)	119 (30.3)	
Combination of 2 infections	48 (52.8)	43 (47.3)	91 (23.1)	
Combination of 3 infections	37 (52.9)	33 (47.1)	70 (17.8)	
Combination of 4 or more infections	73 (64.6)	40 (35.4)	113 (28.8)	
Missing value = 13				
***Hospitalization prior ART initiation***				***0.322***
No	96 (47.8)	105 (52.2)	201 (49.8)	
Yes	87 (42.9)	116 (57.1)	203 (50.2)	
Missing value = 2				
***Number of admissions prior ART (n = 204)***				***0.727***
Single admission	52 (45.2)	63 (54.9)	115 (57.8)	
Two admission	16 (38.1)	26 (61.9)	42 (21.1)	
Multiple admissions	19 (42.7)	27 (57.1)	46 (22.7)	
***WHO clinical stage at ART initiation (n = 203)***				***0.602***
Stage 1 – Asymptomatic	28 (53.9)	24 (46.2)	52 (21.7)	
Stage 2 – Mild	55 (60.4)	36 (39.6)	91 (37.9)	
Stage 3 – Advanced	50 (55.6)	40 (44.4)	90 (37.5)	
Stage 4 – Severe	3 (42.9)	4 (57.1)	7 (2.9)	

### Presenting infections

The most common presenting HIV symptoms when the child was brought to the health facility for HIV testing were the combination of loss of appetite, weight loss as well as general body weakness, which was seen in 189 (49.1%) of the children. The main presenting infections included persistent cough (46.3%), skin infections (43.3%), swollen glands (30.3%), chronic diarrhea (27.6%), vomiting (27.3%), tuberculosis (23.4%), ear infections (21.7%), oral thrush (20.9%), and pneumonia (3.9%). Of the 406 surveyed children, 120(30.6%) presented with one infection, 90 (22.9%) with two infections, while almost half 183 (46.4%) presented with three or more infections (range 1–8), and 13 caregivers did not respond to the question (see Tables 5 and 6).

**Table 6:** **HIV symptoms and infections at diagnosis among children presenting to primary healthcare facilities**

**Variable**	**Number**	**Percent**
***Presenting HIV symptoms***		
Loss of appetite, weight, and weakness	189	49.09
Loss of appetite and weight	20	5.19
Loss of weight and weakness	33	8.57
Loss of appetite and weakness	11	2.86
Loss of weight	67	17.4
Loss of appetite	27	7.01
Weakness	28	7.27
Other	10	2.6
***Presenting infections***		
Persistent cough	188	46.3
Skin infections	176	43.3
Swollen glands	123	30.3
Chronic diarrhea	112	27.6
Vomiting	111	27.3
Pulmonary tuberculosis	95	23.4
Ear infection	88	21.7
Oral thrush	85	20.9
Pneumonia	16	3.9

Compared to children with single infections, at HIV diagnosis, logistic regression analysis showed that orphaned children were less likely to have multiple infections (OR = 0.83, *p* = 0.013, CI: 0.72–0.96) when compared to non-orphans.

### Hospital admissions

Half (n = 203, 50.2%) of the children were admitted to hospital prior to start of ART, almost two thirds (n = 115, 57.8%) had at least one hospital admission, 42 (21.1%) had two admissions, and 46 (22.7%) had three or more admissions. Hospitalization was significantly associated with multiple infections (OR = 1.27, *p* = 0.004, CI: 1.07–1.51). Children who presented with a combination of three infections were almost three times more likely to be admitted (OR = 2.6, *p* = 0.002, CI: 1.43–4.88) than those with two or fewer infections, while those who presented with four and more infections were two times more likely to be admitted (OR = 1.80, *p* = 0.027, CI: 1.07–3.03) than those with two or fewer infections. Children with a history of hospital admission were less likely to be diagnosed late (OR = 0.94, *p* = 0.037, CI: 0.89–0.99). Logistic analysis showed no association between maternal orphan status and hospitalization.

The percentage of children who had at least one hospital admission after the start of ART decreased significantly; only 35 (8.7%) were admitted compared to 203 (50.2%) prior to ART initiation. The details of hospitalization are shown in Table 5.

At HIV diagnosis, 181 (37.9%) of the children were in WHO clinical stages 2 and 3, 52 (21.7%) were in stage 1, and only 7 (2.9%) were in stage 4. Almost two thirds (n = 143, 59.6%) were either asymptomatic or mildly symptomatic at ART initiation, and 97 (40.4%) presented in advanced clinical stages 3 and 4, while the WHO clinical staging data were missing in 166 of the records (see Table 5). The initial viral load at diagnosis was missing in a number of files because the average time since ART initiation was four years and could not be used to assess the immunity status of the children.

## Discussion

The study found that HIV-infected children accessing primary health facilities experienced a high burden of disease at diagnosis and ART initiation. The majority were brought to health facilities for ART follow-up on the day of the survey by their mothers, but because of the high prevalence of orphanhood in this study setting, with 45.2% maternal orphans, children were also brought to health facilities by grandparents, aunts, uncles, and older siblings. Other studies in Sub-Saharan Africa also reported a high prevalence of orphanhood among undiagnosed HIV-infected children; Madiba and colleagues reported a prevalence of 40.9% in an urban study setting [11], Ferrand et al. reported a prevalence of 39% in rural Zimbabwe [26], while Nyandiko and colleagues reported a much higher prevalence (59%) also in an urban setting in Kenya [27]. As reported in many studies in African countries [27-29], grandmothers take the responsibility to care for orphans. In this study, half (50.3%) of the maternal orphans were cared for by their grandparents, while under 4% were cared for by their fathers. There is evidence that fathers of maternal orphaned children do not see their children regularly [30,31]. In this study, for maternal orphaned children whose fathers were alive, only 4% saw their fathers regularly.

Late presentation with undiagnosed perinatal HIV infection was common among children in the primary healthcare facilities where we conducted the survey. Our findings reflect the situation in other studies that were conducted in Sub-Saharan Africa, which found that undiagnosed perinatally infected children present late for treatment and care [2-4,32-35]. The mean age of the children at HIV diagnosis was 4.8 years, and the mean age at ART initiation was 5.6 years. Around half the children were older than five years when they were diagnosed and initiated ART, indicating that children receiving ART in these facilities delayed accessing and initiating ART. These findings indicate that there are still difficulties and barriers in early diagnosis of HIV-infected children in resource-limited settings [32,33,36]. The data showed significant differences between the mean diagnosis and ART initiation ages of maternal orphans and non-orphaned children at univariate analysis. The absence of a biological mother was associated with late and poor access to HIV prevention and treatment, compared to non-orphans; orphaned children were more likely to be diagnosed late and initiate ART late. At multiple regression analysis, only age of ART initiation was significantly associated with orphanhood status. Data from Sub-Saharan Africa show that older survivors among perinatally infected children were mostly orphans diagnosed routinely after recurrent infections [37].

When asked for the reasons for seeking HIV testing, the majority (79%) of primary caregivers reported that the child was severely or chronically ill. As already mentioned, less than half of the children were initiated with ART aged below five years, an indication of low testing of children through the PMTCT program and PITC. In rural South Africa, the uptake of PMTC remains substantially lower than in urban communities. As well as that, services for testing children are limited, while primary caregivers are reluctant to have their children tested [6,9,11,38]. The fact that grandparents were the primary caregivers of half of the maternal orphaned children might have contributed to the delay in seeking HIV testing for the children due to grandparents being ill-informed about HIV symptoms as well as the transmission of HIV from mother to child [32]. Our data showed that primary caregivers of maternal orphans were less educated than those of non-orphans.

Even though the majority of the primary caregivers of orphaned children reported that the child’s biological mother died of AIDS-related illnesses, none sought HIV testing until the child was severely and chronically ill from multiple infections. In this study and others, non-orphaned children of HIV positive biological mothers receiving ART also presented late for HIV diagnosis [6,9,39]. The stigma associated with HIV remains a problem for many primary caregivers who are afraid of positive HIV results for their children. Even though primary caregivers have seen positive outcomes of ART in adult family members and through personal experiences from HIV-infected biological mothers, HIV-related stigma continues to be a barrier to treatment for perinatally infected children [32,33].

In primary health facilities in South Africa, the WHO clinical staging is used to assess children for eligibility for ART initiation in the absence of CD4 count results [24]. The immunologic status of the children at diagnosis and ART initiation was unexpected because literature shows that often older undiagnosed HIV-infected children are severely immunocompromised at diagnosis [32,3,40,11]. We found that almost two-thirds of the children were either asymptomatic or mildly symptomatic and were in clinical stages 1 and 2, while 40.4% presented in advanced clinical stages 3 and 4. It is, however, concerning that the WHO clinical staging data were missing in 166 of the children’s records; therefore, results presented here may not be a true reflection of the immunological status of all the children.

Nevertheless, the available data suggest that the immunologic status of the children was relatively good. We also found no significant difference in the immunologic status of orphaned and non-orphaned children. Data from other studies reported similar findings; studies which compared the WHO clinical staging of rural and urban undiagnosed HIV-infected children in South Africa found less advanced disease among children in rural settings compared to those in urban settings [34]. The relatively healthy immunological status of the children in rural settings should be further investigated; nonetheless, it is an indication of the ability of perinatally infected children to survive for a longer period of time without ART even in rural poor-resource settings [18]. Furthermore, almost half of the children were older than five years at HIV diagnosis, and available data show that a median age of over four years suggests an existence of a population of medium and slow progressors of children [32,33].

Even though the majority of the children were mildly symptomatic, we observed a high burden of infections; over three quarters (69.4%) presented with multiple infections prior to diagnosis and initiation of ART. The main presenting infections included persistent cough, skin infections, swollen glands, chronic diarrhea, vomiting, TB, ear infections, oral thrush, and pneumonia. Close to half (46.4%) presented with three or more infections with a range of 1–8 infections, and only a third (30 5%) presented with one infection. Similar findings of a high burden of infections were reported in previous studies [11,26,27]. We found that being orphaned was not associated with presenting with multiple infections. Though the prevalence of TB among adults in South Africa is high, TB was seen in less than a quarter (23.4%) of the children in this study. The prevalence was less than the 41% reported in a previous study conducted with children in an urban setting in South Africa [11]. Low prevalence (26%) of TB reported in Kenya was attributed to under-diagnosis of TB in children in resource-limited settings [27].

The study further showed that presenting with multiple infections was associated with hospital admission. Half of the children were admitted to hospital prior to diagnosis and start of ART, and those who had multiple infections were three times more likely to be admitted than those with less than two infections. We also found high incidence (43.3%) of multiple hospital admissions among the children. Hospitalization was further associated with the age of diagnosis; children admitted to hospital were less likely to be diagnosed late than those who were never admitted. A Zambian study that reported multiple admissions among children showed that older children had fewer hospital admissions compared to younger children [35], while data from South Africa showed that older children were less likely to be admitted [11]. The current study findings have implications for identifying undiagnosed perinatally infected children at the primary healthcare level; there is evidence that the majority of these children are more likely to be identified during routine hospital screening [3]. Hospitals are more likely to offer sick children PITC and have a higher index of suspicion for HIV infection among undiagnosed HIV-infected children than primary healthcare facilities. It is therefore important that routine PITC is provided to older children in primary healthcare facilities. It is also important to increase awareness among healthcare workers of the high prevalence of HIV infection among older children, and about the benefits of early identification and treatment of HIV infection [38].

The percentage of children who had at least one hospital admission after ART initiation decreased significantly; less than 10% of the children were admitted compared to half prior to ART initiation. Similar findings in other studies showed that hospitalization rates decreased over time after initiation of ART [41].

The study is subject to several limitations, particularly recall bias. The study did not depend on the caregivers to provide information that could be extracted from the children’s records. We conducted semi-structured interviews with primary caregivers and reviewed the children’s records to extract data from laboratory results, as well as some demographic data such as age at start of ART treatment. However, the study depended on the primary caregivers to report the number and types of infections the child presented with prior to the start of ART; the caregivers might have under- or over-reported these conditions. In addition, data on hospital admission was also provided by the primary caregivers and was subject to over- and under-reporting. We also could not provide reasons for hospitalization because those kinds of data are kept in the inpatient records at the hospital. Although majority (91%) of the caregivers reported that the cause of death of the biological mothers of orphaned children was AIDS-related, this could not be confirmed with any clinical data. However, research has shown that up to a third of perinatally infected children are slow progressors with a median survival of 10 years [17]. All the biological mothers of non-orphaned children reported that they were HIV positive and were receiving treatment at the time of data collection. The HIV status of the biological mothers was self-reported and could also not be confirmed with clinical data.

Other limitations of the study were the high treatment defaulter and missed appointment rates observed during the data collection, which resulted in high numbers of children recorded in the facility registers but not included in the survey. The small sample size might suggest low survival rates of slow progressors in rural and poor settings, but this is not the case. We found that in the rural sub-districts, traditional or cultural community celebrations resulted in patients missing their treatment appointment dates, while in the urban district the number of children infected with HIV who missed treatment appointment dates was much higher among patients from the informal settlements that are often referred to as slums. The study is reliable, as the sample was representative of children seeking HIV care and because it was conducted in primary health facilities where HIV care is provided, making the findings generalizable to poor-resource settings. In addition, the high prevalence of orphans also made it possible for us to compare the burden of diseases between orphans and non-orphans.

## Conclusions

The study found that the health of maternal orphaned children was more compromised than non-orphans. They experienced delayed access to HIV treatment and were also likely to be cared for by grandparents who had no education and who depended on the pension grant for source of income. However, the high burden of diseases increased the risks of being hospitalized for all undiagnosed HIV-infected children. The high prevalence of orphanhood among undiagnosed HIV-infected children suggests high mortality of HIV-infected women in these poor-resource communities, and should inform the strengthening of PMTCT programs to increase coverage for all pregnant women. Though literature shows high prevalence of HIV amongst older children in Sub-Saharan Africa, the small total sample size in this study, which included 34 health facilities, suggests missed opportunities for HIV testing of older children in primary health facilities. On the other hand, the sample size could have been influenced by the high defaulter rate or missed appointments observed during the survey. It is important that health workers understand community-related factors that influence treatment defaulter and reasons for missed appointments in order to develop appropriate adherence counseling messages and also tailor the provision of services to the needs of the communities.

Half of the children were admitted to hospital prior to diagnosis and start of ART, and the risk of hospitalization was increased for those who had multiple infections. Then again, hospitalization increased the likelihood of being diagnosed early, most likely because of better screening for HIV when children get admitted to hospital. Perinatally infected children getting delayed access to HIV treatment suggests that primary caregivers are reluctant to test their children for HIV. This is a major constrain to the routine testing and counseling and initiation of ART at the primary healthcare level that is currently being implemented in South Africa. Therefore, there is a need to strengthen the program by identifying the caregivers’ barriers to pediatric HIV testing in order to develop interventions to address these barriers. Stigma and discrimination have been identified major barriers to HIV testing for adults and children, therefore interventions to reduce stigma and discrimination are essential for the success of the implementation of routine HIV testing and counseling in primary health facilities. There is also a need to increase the community and healthcare providers’ awareness about undiagnosed HIV among older children and provide education about benefits of early diagnosis and treatment of HIV infection among this population. Older people caring for undiagnosed HIV infected children in rural settings with limited resources for early diagnosis of children lack the necessary knowledge to understand mother-to-child transmission of HIV. It is crucial that specific strategies targeting elderly caregivers are developed in order to identify HIV infected undiagnosed orphaned children for early treatment and care.

## Additional file

Additional file 1:
**Multilingual abstracts in the six official working languages of the United Nations.**
