# Supplementary material for: The burden of disease on HIV-infected orphaned and non-orphaned children accessing primary health facilities in a rural district with poor resources in South Africa: a cross-sectional survey of primary caregivers of HIV-infected children aged 5–18 years
Source: Infect Dis Poverty. 2015 May 4;4:18. doi: 10.1186/s40249-015-0049-x (PMC4423522; doi:10.1186/s40249-015-0049-x)
Supplement: Additional file 1: — Multilingual abstracts in the six official working languages of the United Nations. [file 40249_2015_49_MOESM1_ESM.pdf]

Translation of the abstract into the six official working languages of the United Nations

عبء المرض على المصابين بفيروس الإيدز من الأطفال الأيتام وغير الأيتام الذين يستخدمون مرافق الرعاية الصحية الأولية في المناطق القروية معدمة الموارد بجنوب أفريقيا: دراسة مستعرضة لمقدمي الرعاية الأساسيين للمصابين بفيروس الإيدز من الأطفال بأعمار من 5 إلى 18 عامًا

ماتيلدا موكجاتل، سفيو ماديبا

نبذة مختصرة

خلفية: تنصح منظمة الصحة العالمية (WHO) بأن يتم تقديم الفحوصات والاستشارات الخاصة بفيروس الإيدز المقدمة من متعهدين (PITC) والمعممة كجزء من الرعاية الصحية الاعتيادية، وذلك لزيادة فرصة تلقي الأطفال المصابين بفيروس الإيدز للعلاج. ما يحدث بالفعل هو أن تقديم الفحوصات الخاصة بفيروس الإيدز يكون مرهونًا بظهور الأعراض المصاحبة لفيروس الإيدز على الأطفال. وبالتالي، فإنه يتم تشخيص فيروس الإيدز في مرحلة متأخرة من الطفولة بعد حدوث حالات عدوى متكررة ومزمنة. لقد قمنا بدراسة الأسباب التي أفصح عنها مقدمو الرعاية الأساسيين للأطفال فيما يتعلق بالسعي لإجراء فحوصات فيروس الإيدز للأطفال الذين تتراوح أعمارهم بين 5 و18 عامًا، كما قمنا بتحديد حالة اليتم عند الأطفال والمقارنة بين الملف الإكلينيكي وعبء المرض الذي يقع على الأيتام وغير الأيتام.

الوسائل: تناولت هذه الدراسة المستعرضة مقدمي الرعاية الأساسيين للأطفال المصابين بفيروس الإيدز والمتلقين لعلاجات الفيروسات القهقرية (ART) في مشفيين مجتمعيين و34 منشأة رعاية صحية في منطقة قروية بمحافظة مبومالانجا في جنوب أفريقيا.

النتائج: تألفت العينة من 406 فرد من مقدمي الرعاية الأساسيين: منهم 319 فرد (78.6%) قاموا بإحضار أطفالهم إلى منشأة الرعاية الصحية لإجراء فحوصات فيروس الإيدز بسبب حالات العدوى المزمنة والمتكررة. وكان نصف عدد الأطفال تقريبًا في العينة (n=183، 45.1%) يتيم الأم، و128 طفل (31.5%) يتيم الأب، و73 طفل (39.9%) يتيم الأبوين. وقد أفاد التحليل أحادي المتغير بأن أيتام الأم هم الأطفال الأكبر سنًا على الأرجح (OR=2.57، p=0.000، CI: 1.71–3.84) ويتم تشخيص المرض في مرحلة متأخرة عندهم (OR=2.48، p= 0.009، CI: 1.26–4.88) ويبدأون في تلقي علاجات الفيروسات القهقرية في مرحلة متأخرة أيضاً (OR=2.5، p=0.007، CI: 1.28–4.89) بالمقارنة مع الأطفال غير الأيتام. كما خلصت الدراسة إلى وجود عبء أكبر لحالات العدوى بين الأطفال قبل تشخيص فيروس الإيدز؛ حيث يعاني 274 (69.4%) طفل من حالات عدوى مضاعفة. وقد أظهر الانحدار اللوجستي أن العمر عند بدء تلقي علاجات الفيروسات القهقرية (aOR=1.19، p=0.000، CI: 1.10–1.29) والوقت المستغرق في تلقي علاجات الفيروسات القهقرية (aOR=2.30، p=0.000، CI: 1.45–3.64) يرتبطان بدرجة كبيرة بحالة اليتم. فقد دخل نصف عدد الأطفال (n=203، 50.2%) إلى المشفى قبل بدء تلقي علاجات الفيروسات القهقرية، كما ارتبطت فترة الإقامة في المستشفى ارتباطًا وثيقًا بحالات العدوى المضاعفة (OR=1.27، p=0.004، CI: 1.07–1.51).

الخلاصة: توصلّت الدراسة إلى وجود اكتشاف متأخر لعدوى فيروس الإيدز غير المشخّصة خلال فترة ما قبل الولادة وشيوع اليتم بين الأطفال. ويعاني الأطفال أيتام الأم مشاكل صحية أكبر بالمقارنة بغير الأيتام. كما ينبغي دعم توفير الفحوصات والاستشارات الخاصة بفيروس الإيدز المقدمة من متعهدين (PITC) لزيادة وعي المجتمع بشأن إصابات فيروس الإيدز غير المشخصة بين الأطفال الأكبر سنًا لتشجيع مقدمي الرعاية الأساسيين على قبول إجراء اختبارات تشخيص الإصابة بفيروس الإيدز لدى الأطفال. ومن الضروري أن يتم تطوير استراتيجيات تستهدف كبار السن من مقدمي الرعاية الأساسيين لتحديد الأيتام المصابين بالعدوى قبل الولادة ولم يتم تشخيصهم حتى يحصلوا على العلاج والرعاية المبكرة.

Translated from English version into Arabic by Amal_Ibrahim, through

TRANSLATORS WITHOUT BORDERS

南非边远的农村地区感染艾滋病病毒的孤儿和非孤获得基本医疗的疾病负担：针对5-18岁艾滋病病毒感染者主要陪护人的横断面研究

Mathildah Mokgatle, Sphiwe Madiba

摘要

引言：世界卫生组织提出，在艾滋病流行区，应向感染艾滋病病毒的少年儿童提供艾滋病毒检测和咨询服务，并把它当做一项正规医疗标准来提高艾滋病就医率。实际上，能否进行艾滋病病毒检测主要取决于儿童是否出现相关艾滋病症状。少年儿童只有在出现反复和长期感染时才会进行艾滋病病毒诊断。我们调查了 5-18 岁孩子的主要陪护人，询问了他们给孩子进行艾滋病病毒检测的原因，并对孤儿和非孤儿的诊疗记录和疾病负担进行比较。

方法：采取横断面调查研究的方法，在南非普马兰加省的边远地区的两个社区医院和 34 所初级卫生保健机构中，对感染艾滋病病毒并接受抗病毒治疗的少年儿童的主要陪护人进行调查。

结果：共406人接受了调查，其中有319人（76%）是因为孩子长期反复感染后才去医疗机构进行艾滋病病毒检测的。近一半的少年儿童（45.1%, 183人）是失母孤儿，128个孩子（31.5%）是失父孤儿，还有73个孩子（39.9%）失去了双亲。单因素分析表明，与非孤患者相比，感染艾滋病病毒失母孤儿的年龄较大（OR=2.57, p=0.000, CI: 1.71–3.84），送诊较晚(OR=2.48, p= 0.009, CI: 1.26–4.88)，开始抗病毒治疗的时间也较迟(OR=2.5, p=0.007, CI: 1.28–4.89)。还有一个比艾滋病病毒诊断更沉重的负担是274名少年儿童（69.4%）呈现多重感染。多元因素Logistic回归分析显示，艾滋病感染者的孤儿身份与接受艾滋病病毒检测时的年龄(aOR=1.19, p=0.000, CI: 1.10–1.29)和时间(aOR=2.30, p=0.000, CI: 1.45–3.64)显著相关。有一半的少年儿童（203人，50.2%）是先住院才进行艾滋病抗病毒治疗，住院治疗与多重感染有显著相关性(OR=1.27, p=0.004, CI: 1.07–1.51)。

结论：本研究发现孤儿中存在着未确诊围产期艾滋病毒感染晚期症状和高发病率。失母孤儿比其他儿童的身体抵抗力差。因此，应当加强常规艾滋病病毒咨询检测，提高社会对未确诊的少年儿童中艾滋病病毒感染情况的关注度，并鼓励

其主要陪护人带他们进行艾滋病病毒检测。为保证未确诊围产期感染孤儿早日就医和及时护理，针对年长护理人员的培训策略也至关重要。

Translated from English version into Chinese by Chen Jin, edited by Yin Jian-hai, through

CHINESE CENTER FOR DISEASE CONTROL AND PREVENTION
NATIONAL INSTITUTE OF PARASITIC DISEASES

Poids de la maladie pour les enfants orphelins et non orphelins infectés par le VIH accédant aux structures de santé primaires d'un district rural à faibles ressources en Afrique du Sud : étude transversale auprès d'adultes responsables d'enfants de 5 à 18 ans infectés par le VIH

Mathildah Mokgatle, Sphiwe Madiba

Résumé
Contexte : L'Organisation mondiale pour la santé recommande qu'en cas d'épidémie de VIH généralisée, des services de conseil et de dépistage du VIH à l'initiative du soignant soient proposés dans le cadre des soins standard, afin de faciliter l'accès des enfants infectés par le VIH au traitement. En pratique, le VIH est recherché lorsque les enfants présentent des symptômes qui lui sont liés, de sorte que le diagnostic est posé chez des enfants déjà grands, à la suite d'infections récidivantes et chroniques. Nous avons étudié les raisons invoquées par les parents ou tuteurs ayant demandé le dépistage du VIH chez des enfants âgés de 5 à 18 ans, déterminé le statut familial (orphelins ou non) des enfants et comparé le profil clinique et le poids de la maladie pour les orphelins et les non orphelins.
Méthodes : Nous présentons une étude transversale des adultes responsables d'enfants infectés par le VIH recevant un traitement antirétroviral (TARV) dans 2 hôpitaux locaux et 34 centres de soins de santé primaires d'un district rural dans la province de Mpumalanga, en Afrique du Sud.
Résultats : L'échantillon comprenait 406 adultes responsables : 319 (78,6 %) avaient amené l'enfant au centre de santé pour un dépistage du VIH en raison d'infections chroniques et récidivantes. Près de la moitié (n=183, 45,1 %) des enfants étaient orphelins de mère, 128 (31,5 %) orphelins de père et 73 (39,9 %) orphelins de leurs deux parents. Une analyse univariée a montré une tendance significative des orphelins de mère à être plus âgés (OR=2,57, p=0,000, IC : 1,71–3,84), à être diagnostiqués plus tardivement (OR=2,48 , p= 0,009, IC : 1,26–4,88) et à commencer le TARV plus tard (OR=2,5, p=0,007, IC : 1,28–4,89) que les non orphelins. La charge infectieuse était importante parmi les enfants avant le diagnostic de VIH : 274 (69,4 %) présentaient des infections multiples. Une régression logistique multiple a montré que l'âge de début du TARV (aOR=1,19 , p=0,000, IC : 1,10–1,29) et la durée du TARV (aOR=2,30 , p=0,000, IC : 1,45–3,64) étaient significativement associés au statut d'orphelin. La moitié (n=203, 50,2 %) des enfants avaient été admis à l'hôpital avant la mise en place du TARV et l'hospitalisation était significativement associée à des infections multiples (OR=1,27 , p=0,004, IC : 1,07–1,51).
Conclusion : L'étude a mis en évidence la présentation tardive d'infections périnatales par le VIH non diagnostiquées et une forte prévalence des orphelins parmi les enfants. L'état de santé des orphelins de mère était plus compromis que celui des non orphelins. Il serait bon d'intensifier le conseil et le dépistage du VIH à l'initiative du soignant, afin de sensibiliser les populations aux infections non diagnostiquées par le VIH parmi les grands enfants et d'encourager les parents ou les tuteurs à accepter le dépistage du VIH chez les enfants. Il est crucial que des stratégies visant les parents et tuteurs âgés soient mises au point, afin d'identifier les orphelins infectés à la naissance et non diagnostiqués en vue de leur traitement et des soins nécessaires.

Translated from English version into French by Suzanne Assenat, through

TRANSLATORS
WITHOUT BORDERS

Бремя заболевания на ВИЧ-инфицированных детях и детях-сиротах, имеющих доступ к центрам первичной медицинской помощи в сельской местности с ограниченными ресурсами в ЮАР: перекрестное исследование ответственных опекунов ВИЧ-инфицированных детей в возрасте от 5 до 18 лет

Матильда Мокгатле, Сфиве Мадиба

Резюме
Базовая проблематика: В условиях генерализованных эпидемий ВИЧ Всемирная организация здоровья (ВОЗ) рекомендует включать тестирование на ВИЧ и консультирование по вопросам ВИЧ по инициативе поставщиков

медицинских услуг в стандартный набор медобслуживания с целью повысить доступ к лечению ВИЧ-инфицированных детей. На практике тестирование на ВИЧ проводится на основании наличия симптомов у детей; таким образом, диагноз на ВИЧ-инфекцию ставится в позднем детстве, вслед за периодическими и хроническими инфекциями. Мы исследовали причины, приведенные ответственными опекунами при обращении за тестированием на ВИЧ для детей в возрасте от 5 до 18 лет, определили статус сиротства детей и сравнили клинические данные и бремя заболевания на детях-сиротах и детях, имеющих родителей.
Методы: Мы провели перекрестное исследование ответственных опекунов ВИЧ-инфицированных детей, получающих антиретровирусную терапию (АРТ) в двух больницах общего профиля и 34 центрах первичной медицинской помощи в сельской местности в провинции Мпумаланга, ЮАР.
Результаты: Выборка состояла из 406 ответственных опекунов: 319 (78,6%) из них привели ребенка в медицинский центр в связи с периодическими и хроническими инфекциями. Почти половина (n=183, 45,1%) из детей не имели матерей, 128 (31,5%) не имели отцов и 73 (39,9%) не имели обоих родителей. Одномерный анализ показал, что дети без матерей были значительно старше (ОШ=2,57, *p*=0,000, ДИ: 1,71-3.84), позднее диагностировались (ОШ=2,48, p=0,009, ДИ:1,26-4,88) и позднее начинали курс АРТ (ОШ:2,5, p=0,007, ДИ: 1,28-4,89), чем дети, имеющие обоих родителей. До момента выявления ВИЧ-инфекции среди детей наблюдались высокие показатели распространенности инфекций: 274 ребенка (69,4%) обратились с многочисленными инфекциями. Многопараметрическая логистическая регрессия показала значительную связь между возрастом назначения АРТ (СОШ: 1,19, p=0,000, ДИ: 1,10-1,29), продолжительностью курса АРТ (СОШ: 2,30, p=0,000, ДИ: 1,45-3,64) и статусом сиротства. Половина детей (n=203, 50,2%) были госпитализированы до начала курса АРТ, и госпитализация была значительно связана с многочисленными инфекциями (ОШ: 1,27, p=0,004, ДИ: 1,07-1,51).
Заключение: Исследование показало позднее обращение с невыявленной перинатальной ВИЧ-инфекцией и высокую распространенность сиротства среди детей. Здоровье детей без матерей подвержено большему риску, чем детей, имеющих обоих родителей. Необходимо усилить рутинное тестирование и консультирование по инициативе тех, кто предоставляет услуги, с целью повышения информированности населения о невыявленной ВИЧ-инфекции среди детей старшего возраста и поощрения ответственных опекунов на разрешение проведения тестирования на ВИЧ у детей. Важной задачей является разработка стратегий для пожилых ответственных опекунов с целью выявления перинатально-инфицированных сирот для раннего лечения и ухода.

Translated from English version into Russian by Nurangiz Khodzharova, through

TRANSLATORS
WITHOUT BORDERS

La carga de la enfermedad sobre niños huérfanos y no huérfanos infectados con VIH con acceso a las instalaciones de salud básica en un distrito rural de escasos recursos en Sudáfrica: Un estudio transversal realizado entre cuidadores principales de niños de 5 a 18 años de edad infectados con VIH.

Mathildah Mokgatle,
Sphiwe Madiba

Resumen

Antecedentes: La Organización Mundial de la Salud recomienda que, en epidemias generalizadas de VIH, se ofrezcan pruebas de detección y asesorías sobre el VIH (APV) por iniciativa del proveedor de servicios de salud como parte del estándar de atención normal para aumentar el acceso de los niños infectados con VIH a un tratamiento apropiado. En la práctica, la prueba de detección de VIH se aplica a los niños que presentan síntomas relacionados con el VIH; en consecuencia, el diagnóstico de presencia del VIH ocurre en las últimas etapas de la niñez, después de que se presentan infecciones recurrentes y crónicas. Investigamos las razones informadas por los cuidadores principales para buscar la aplicación de la prueba de detección de VIH a niños de 5 a 18 años de edad, determinamos la situación de orfandad de los niños y comparamos el perfil clínico y la carga de la enfermedad para quienes son huérfanos y quienes no lo son.
Métodos: Este fue un estudio transversal que se llevó a cabo entre cuidadores principales de niños infectados con VIH que tienen acceso a la terapia antirretroviral (TARV) en dos hospitales comunitarios y 34 centros de atención a la salud básicos en un distrito rural de la provincia de Mpumalanga, Sudáfrica.
Resultados: La muestra constó de 406 cuidadores personales: 319 (78.6%) llevaron al menor al centro de salud para someterlo a una prueba de VIH debido a la presencia de infecciones crónicas y recurrentes. Casi la mitad (n=183, 45.1%) de los niños eran huérfanos de madre, 128 (31.5%) eran huérfanos de padre y 73 (39.9%) eran huérfanos por partida doble. Un análisis univariado demostró que los niños huérfanos de madre tenían una probabilidad significativamente más alta de ser mayores (OR=2.57, *p*=0.000, IC: 1.71–3.84), de tener un diagnóstico tardío (OR=2.48, *p*= 0.009, IC: 1.26–4.88) y de empezar la TARV después (OR=2.5, *p*=0.007, IC: 1.28–4.89) que quienes no eran huérfanos. Se encontró una carga de infección alta entre los niños antes de ser diagnosticados como positivos al VIH; 274 (69.4%) presentaban varias infecciones. La regresión logística multinomial

demostró que la edad de inicio de la TARV (aOR=1.19, p=0.000, IC: 1.10–1.29) y la duración de la TARV (aOR=2.30, p=0.000, IC: 1.45–3.64) estaban asociados de manera significativa con la condición de orfandad. La mitad (n=203, (50.2%) de los niños fueron admitidos en el hospital antes de iniciar la TARV y la hospitalización estaba fuertemente relacionada con la presencia de varias infecciones (OR=1.27, p=0.004, IC: 1.07–1.51).

Conclusión: El estudio encontró presentación tardía de casos con infección perinatal por VIH sin diagnosticar y una alta prevalencia de orfandad entre los niños infectados. La salud de los niños huérfanos de madre estaba más afectada que la de quienes no eran huérfanos. Debe fortalecerse la aplicación de APV de rutina para aumentar el conocimiento de la comunidad sobre la presencia no diagnosticada del VIH entre los niños mayores y para motivar a los cuidadores principales a que acepten que los niños sean sometidos a pruebas de detección del VIH. Es crucial que se desarrollen estrategias dirigidas a los cuidadores de los niños mayores para identificar huérfanos infectados perinatalmente y no diagnosticados para darles un tratamiento y atención oportunos.

Translated from English version into Spanish by Silvia Sassone, through

TRANSLATORS
WITHOUT BORDERS
